# Cytoprotective Roles of a Novel Compound, MHY-1684, against Hyperglycemia-Induced Oxidative Stress and Mitochondrial Dysfunction in Human Cardiac Progenitor Cells

**DOI:** 10.1155/2018/4528184

**Published:** 2018-05-30

**Authors:** Woong Bi Jang, Ji Hye Park, Seung Taek Ji, Na Kyung Lee, Da Yeon Kim, Yeon Ju Kim, Seok Yun Jung, Songhwa Kang, Shreekrishna Lamichane, Babita Dahal Lamichane, Jongseong Ha, Jisoo Yun, Hyung Ryong Moon, Sang Hong Baek, Hae Young Chung, Sang-Mo Kwon

**Affiliations:** ^1^Laboratory for Vascular Medicine and Stem Cell Biology, Medical Research Institute, Department of Physiology, School of Medicine, Pusan National University, Yangsan 50612, Republic of Korea; ^2^Convergence Stem Cell Research Center, Pusan National University, Yangsan, Republic of Korea; ^3^Molecular Inflammation Research Center for Aging Intervention, College of Pharmacy, Pusan National University, Busan, Republic of Korea; ^4^Laboratory of Medicinal Chemistry, College of Pharmacy, Pusan National University, Busan, Republic of Korea; ^5^Laboratory of Cardiovascular Disease, Division of Cardiology, School of Medicine, The Catholic University of Korea, Seoul, Republic of Korea; ^6^Research Institute of Convergence Biomedical Science and Technology, Pusan National University Yangsan Hospital, Yangsan, Republic of Korea

## Abstract

Diabetic cardiomyopathy (DCM) is tightly linked to heart disorders and dysfunction or death of the cardiomyocytes including resident cardiac progenitor cells (CPCs) in diabetic patients. In order to restore loss of function of resident or transplanted CPCs, much research has focused on novel therapeutic strategies including the discovery of novel function-modulating factors such as reactive oxygen species (ROS) scavengers. Here, we developed and defined a novel antioxidant, MHY-1684, for enhancing the angiogenic potential of CPCs against ROS-related DCM. Short-term treatment with MHY-1684 restored ROS-induced CPC cell death. Importantly, MHY-1684 decreased hyperglycemia-induced mitochondrial ROS generation and attenuated hyperglycemia-induced mitochondrial fragmentation. We observed that the activation process of both Drp1 (phosphorylation at the site of Ser616) and Fis-1 is drastically attenuated when exposed to high concentrations of D-glucose with MHY-1684. Interestingly, phosphorylation of Drp1 at the site of Ser637, which is an inhibitory signal for mitochondrial fusion, is restored by MHY-1684 treatment, suggesting that this antioxidant may affect the activation and inhibition of mitochondrial dynamics-related signaling and mitochondrial function in response to ROS stress. In conclusion, our finding of the novel compound, MHY-1684, as an ROS scavenger, might provide an effective therapeutic strategy for CPC-based therapy against diabetic cardiomyopathy.

## 1. Introduction

Diabetes mellitus (DM), commonly called as diabetes, is a common public health problem worldwide. The most severe problems associated with diabetes are the various DM-related complications such as cardiovascular diseases, renal diseases, neuropathy, and diabetic nephropathy. In general, the primary cause of diabetes is a dysregulation of the blood glucose level due to a malfunction in insulin secretion [1]. This malfunction is caused by the destruction of pancreatic beta cells in type I diabetes and insulin resistance in type II diabetes. Regardless of the type, diabetes is categorized as a disease where blood glucose is elevated, and this eventually leads to hyperglycemia, which is a major cause of cardiovascular disease. In clinical settings, it has been reported that the death ratio of diabetes patients with cardiovascular disease is much higher than normal patients with cardiovascular disease [2, 3]. Furthermore, hyperglycemia causes additional complications such as neuropathy, stroke, diabetic ketoacidosis, and a hyperosmolar hyperglycemic state [4, 5].

Diabetic cardiomyopathy (DCM) leads to heart disorders and dysfunction or death of the resident cardiomyocytes in diabetic patients. DCM is characterized by an altered lipid composition and mitochondrial dysfunction in diabetic hearts [6], which develop from various causative factors such as oxidative stress, mitochondrial dysfunction, endothelial cell loss, inflammation, autophagy, and mitophagy [7, 8]. In order to reduce the risk of heart failure, DCM needs to be controlled via reducing hyperglycemia and improving the cellular function of cardiomyocytes or resident cardiac progenitor cells (CPCs). In a clinical setting, hyperglycemia by induced DCM affects CPC viability and the angiogenic potential of resident CPCs or transplanted CPCs [9]. Therefore, identifying novel factors that have cytoprotective effects against hyperglycemia is a prerequisite for cell-based therapies in the treatment of DCM.

It is generally well known that the overproduction of reactive oxygen species (ROS) has a critical impact on the development of diabetic complications [10]. Excess ROS in tissues gives rise to mitochondrial dysfunction, which eventually causes cell senescence and cell death, as well as the reduced bioactivity of pancreatic cells including tissue stem cells [11, 12]. Several studies have focused on the cytoprotective effect of natural or synthetic compounds as ROS scavengers [13–15]. Recently, we showed that oleuropein-primed endothelial progenitor cells (EPCs) modulate the potential of vascular repair against activating the ROS-induced extracellular signal-regulated kinase 1/2-peroxiredoxin (ERK1/2-Prdx) pathway [15]. Moreover, pretreatment with lycopene attenuates ROS-induced apoptosis in human mesenchymal stem cell (hMSC) through the protein kinase B-manganese superoxide dismutase (AKT-MnSOD) pathway [14].

Accumulating evidence has suggested that mitochondrial dynamics including mitochondrial fission and fusion maintain mitochondrial homeostasis [16]. For example, several research groups have clearly demonstrated the specific disruption of mitochondrial dynamics by fission-related inhibitors including specific targeting of dynamin-related protein 1 (Drp-1), which eventually induces cell death and promotes heart disease [17–19]. Multiple therapeutic strategies have been attempted, including the specific targeting of ROS-induced mitochondrial fragmentation [20], hyperglycemia-related cardiac stem cell homing [21], and hyperglycemia-induced angiogenic capacity of CPCs [9]. Recently, we also reported that hyperglycemia in CPCs alters mitochondrial dynamics and increases the expression of fission-related proteins, including mitochondrial fission 1 protein (Fis1) and Drp1. Moreover, we showed that the specific blockage of glucose transporter 1 (GLUT1) improved cell viability, tube formation, and regulation of mitochondrial dynamics in CPCs [17]. Nevertheless, there are still few reports that identify novel compounds for enhancing CPC bioactivities against DCM.

Thus, the aim of this study is to develop a novel antioxidant compound against hyperglycemia-induced cardiomyopathy and evaluate the potential roles of our compound in oxidative stress, cell death, and mitochondrial dynamics in CPCs.

## 2. Material and Methods

### 2.1. Isolation of Human c-Kit-Positive Cardiac Progenitor Cells (hCPCs^c-kit+^)

hCPCs^c-kit+^ were isolated from human infant-derived heart tissues after surgical procedures, as described in a previously modified protocol [22]. The Ethical Review Board of the Pusan National University Yangsan Hospital, Gyeongsangnam-do, Republic of Korea, approved the protocols. To perform this isolation, the biopsied heart specimens were minced and incubated in 0.2% collagenase type II (Worthington, NJ, USA) at 37°C for 30 min to obtain single cardiac cells. Single cardiac cells were incubated in Ham's F12 media (HyClone, UT) containing 10% fetal bovine serum (FBS, Gibco, CA, USA), 1x penicillin/streptomycin (P/S, Welgene, Daegu, Republic of Korea), 2.5 U human erythropoietin (hEPO, R&D Systems, Minneapolis, MN, USA), 5 *μ*g basic human recombinant fibroblast growth factor (bFGF, PeproTech, Rocky Hill, NJ, USA), and 0.2 mM glutathione (Sigma-Aldrich, St. Louis, CA, USA). When single cardiac cells were grown to a high enough confluence for sorting, single cardiac cells were conjugated to the c-kit primary antibody (Santa Cruz Biotechnology, Santa Cruz, CA, USA) and sorted by magnetic activated cell sorting (MACS).

### 2.2. Measurement of Peroxynitrite (ONOO^−^) Scavenging Activity

ONOO^−^ (Eugene, Oregon, USA) scavenging was measured using monitoring the oxidation of dihydrorhodamine 123 (DHR 123, Eugene, Oregon, USA) by modifying the method [23]. ONOO^−^ scavenging by the oxidation of DHR 123 was measured on a microplate fluorescence spectrophotometer FL 500 (BioTek Instruments, USA) with excitation and emission wavelengths of 485 nm and 530 nm, respectively, at room temperature. The background and final fluorescent intensities were measured at 5 min after treatment with or without SIN-1 (final concentration, 10 mM) or authentic ONOO^−^ (final concentration, 10 mM) in 0.3 N sodium hydroxide. Oxidation of DHR 123 by decomposition of SIN-1 (Sigma-Aldrich, St. Louis, CA, USA) gradually increased, whereas authentic ONOO^−^ rapidly oxidized DHR 123 with its final fluorescent intensity being stable over time.

### 2.3. hCPC^c-kit+^ Cultures and MHY-1684

hCPCs^c-kit+^ were maintained in HAM's F12 culture media at 37°C in humidified 5% CO_2_. D-(+) glucose (Sigma-Aldrich, St. Louis, CA, USA) was added to the normal culture media. MHY-1684 was dissolved in DMSO (100 mM) and further diluted for different concentrations. Same concentration of DMSO was added to the control group of all experiments. MHY-1684 was used as an antioxidant. hCPCs were treated with D-(+) glucose and MHY-1684 at various concentrations.

### 2.4. Cell Viability Assay

Cell viability was measured using the WST Kit (Ez-Cytox, Dail Lab, Seoul, Republic of Korea). hCPCs^c-kit+^ were seeded on 96-well plates with D-(+) glucose and MHY-1684 at various concentrations. Culture plates were incubated for 24, 48, and 72 h. After incubation, the culture medium with D-(+) glucose and MHY-1684 at various concentrations was changed to the WST solution. The plates were then incubated for 1 h, and the absorbance was measured at 450 nm using a spectrophotometer (Tecan, Grodig, Austria).

### 2.5. Western Blot Analysis

Whole cells were lysed with RIPA lysis and extraction buffer (Thermo Fisher Scientific, Waltham, MA, USA) with a protease inhibitor cocktail (Sigma-Aldrich, St. Louis, CA, USA) on ice, and the total protein concentration was quantified using a Bicinchoninic Acid Kit (Thermo Scientific, Rockford, IL, USA). In general, 10–25 *μ*g of total protein was separated by 8–15% sodium dodecyl sulfate polyacrylamide gel electrophoresis, and the proteins were transferred to polyvinylidene fluoride (PVDF, Millipore, Billerica, MA, USA) membranes. The membranes were blocked with 5% skim milk for 1 h at room temperature and incubated with primary antibodies overnight at 4°C. Subsequently, these membranes were incubated with horseradish peroxidase- (HRP-) conjugated secondary antibodies for 1 h at room temperature (Millipore), and protein bands were visualized using the enhanced chemiluminescence reagents (Thermo Fisher Scientific). The following primary antibodies were used: phosphorylated extracellular signal-regulated kinase (p-ERK, Cell Signaling Technology, Danvers, MA, USA), extracellular signal-regulated kinase (ERK, Cell Signaling Technology), phosphorylated protein kinase-B (p-AKT, Cell Signaling Technology), protein kinase-B (AKT, Cell Signaling Technology), AMP-activated protein kinase (AMPK, Cell Signaling Technology), glyceraldehyde-3-phosphate dehydrogenase (GAPDH, Santa Cruz Biotechnology, Santa Cruz, CA, USA), *β*-actin (Santa Cruz), p-Drp1ser^616^ (Cell Signaling Technology), p-DRP1ser^637^ (Cell Signaling Technology), Drp1 (Cell Signaling Technology), Fis1 (Abcam, Cambridge, MA, USA), mitofusin-1 (Mfn-1, Santa Cruz), and optic atrophy 1 (OPA1, Abcam). The following secondary antibodies were used: goat anti-rabbit IgG and goat anti-mouse IgG (Enzo Life Sciences, Farmingdale, USA).

### 2.6. Tube Formation Assay

96-Well plates were coated with 70 *μ*L of Matrigel (BD Biosciences, Franklin Lakes, NJ) and incubated at 37°C for 30 min. hCPCs were seeded in 96-well plates with Matrigel and then incubated for 6 h. After the incubation, total tube length was measured by counting the number of tubes visualized in one microscopic field per well (40x magnification) at least 3 independent replicates using the ImageJ software (free software from National Institutes of Health).

### 2.7. Measurement of Mitochondrial ROS

Mitochondrial ROS was determined using the fluorescent MitoSOX probe (Invitrogen, Carlsbad, CA, USA). hCPCs were incubated in HAM's F12 culture media with 2 *μ*M MitoSOX Red for 30 min at 37°C in a 5% CO_2_ atmosphere. After the incubation, the cells were washed with phosphate-buffered saline (PBS), and the fluorescence was assessed by fluorescence-activated cell sorting (FACS; BD Accuri C6, San Jose, CA, USA). Mitochondrial ROS were analyzed using BD FACSDiva software (BD Biosciences, Bedford, MA).

### 2.8. Cell Death Analysis

To measure apoptotic cell death, hCPCs were detected by FACS (BD Accuri C6) with annexin V/propidium iodide (PI) Apoptosis Detection Kit (BD Biosciences, San Diego, CA, USA). Gently, hCPCs were harvested after the addition of culture media that contained D-(+) glucose and MHY-1684. The cells were then treated with annexin V/PI for at least 15 min in the dark according to the manufacturer's instructions.

### 2.9. Immunofluorescence

Mitochondrial morphology was measured in hCPCs stained with 200 nM MitoTracker Red CMXRos (Molecular Probes, Eugene, OR, USA) with a confocal microscope (Olympus, FV1000, Tokyo, Japan). To determine any alteration in mitochondrial morphology, we measured mitochondrial total length using the ImageJ software.

### 2.10. Statistical Analysis

All experimental results are presented as the mean ± standard deviation (SD) using ANOVA. Comparisons between the two groups were performed using the unpaired Student's *t*-test. A value of *p* < 0.05 was considered statistically significant.

## 3. Results

### 3.1. Development of a Novel Compound, MHY-1684, for Enhancing the Bioactivity of hCPCs

**Figure 1:**
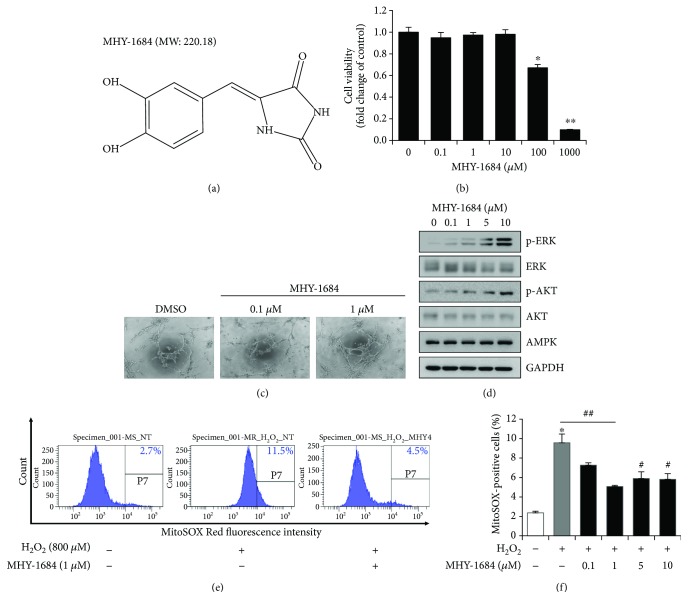
The role of MHY-1684 as an ROS scavenger in hCPCs. (a) The chemical structure of MHY-1684. (b) hCPCs were seeded in 96-well plates and cultured with the indicated concentrations of MHY-1684 for 24 h. Cell viability was assessed using the WST assay. Values are the mean ± S.E.M. ^∗^ *p* < 0.05 and ^∗∗^ *p* < 0.01 as compared with the control group. (c) The tube-forming ability of hCPCs was examined when cells were exposed to MHY-1684 for 24 h. The tubular-like network was evaluated using a Matrigel tube formation assay. (d) After treatment with MHY-1684 for 24 h, the prosurvival-related proteins (ERK-1, AKT-1) were analyzed by Western blotting. (e) hCPCs were pretreated with the indicated concentrations of MHY-1684 for 24 h prior to hydrogen peroxide (H_2_O_2_, 800 *μ*M) treatment. Mitochondrial ROS levels in hCPCs were measured by flow cytometry with MitoSOX Red. (f) Histogram indicates median value ± S.E.M. of MitoSOX-positive cells when exposed to H_2_O_2_. MitoSOX-positive cells increased when exposed to H_2_O_2_; however, when cotreated with MHY-1684, the MitoSOX-positive cells decreased significantly. Values are the mean ± S.E.M. ^∗^ *p* < 0.05 and ^∗∗^ *p* < 0.01 as compared with the control group; ^#^ *p* < 0.05 and ^##^ *p* < 0.01 as compared with the group treated with 25 mM D-(+) glucose for 72 h.

In order to develop a novel compound, we designed a MHY-1684 as an antioxidant compound, which is synthesized based on the kojic acid (Figure 1(a)). Previously, antioxidant effect of kojic acid has been reported [24, 25]. It confirmed the antioxidant effect of MHY-1684 (Supplementary Table 1, Supplementary Materials). To evaluate the cytotoxicity of MHY-1684 on hCPCs, we treated the cells with 0.1, 1, 10, 100 *μ*M, and 1 mM MHY-1684 for 24 h. Cell viability decreased significantly when cells were exposed to more than 10 *μ*M MHY-1684. Therefore, we optimized all experiments using less than 10 *μ*M MHY-1684 (Figure 1(b)). To evaluate the impact of MHY-1684 on hCPC bioactivity, we next investigated the effect of MHY-1684 on hCPC tube-forming ability and cell proliferation, including an investigation into the ERK1/2 and AKT pathway. Treatment of hCPC with MHY-1684 for 24 h significantly increased the expression of p-ERK and p-AKT (Figures 1(c) and 1(d)), as well as enhanced hCPC tube-forming capacity, suggesting that MHY-1684 affects hCPC angiogenic potential and cell proliferation.

### 3.2. Antioxidant Effect of MHY-1684 on H_2_O_2_-Induced ROS in hCPCs

To determine the potential role of MHY-1684 as an antioxidant, we examined mitochondrial ROS generation in response to oxidative stress. Specifically, when hCPCs were exposed to 800 *μ*M of H_2_O_2_, a significant amount of mitochondrial ROS was produced. In contrast, hCPCs pretreated with MHY-1684 displayed a dramatic decrease in the level of mitochondrial ROS, suggesting that MHY-1684 has an inhibitory effect on mitochondrial ROS generation (Figures 1(e) and 1(f)).

### 3.3. Cytoprotective Effect of MHY-1684 on Hyperglycemia-Induced Cell Death in hCPCs

**Figure 2:**
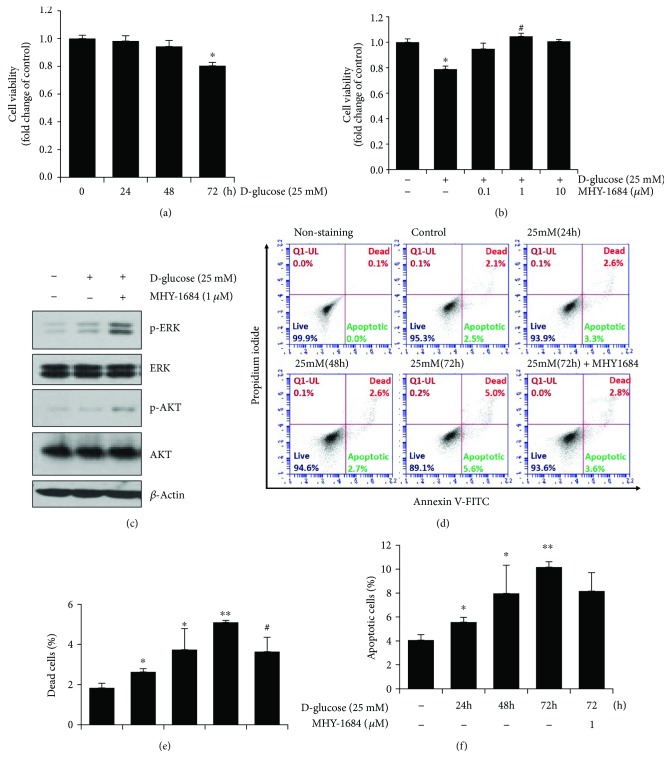
Effect of MHY-1684 on hyperglycemia-induced cell death of hCPCs. (a) hCPCs were treated with 25 mM D-(+) glucose for 0–72 h, and cell viability was subsequently assessed. (b) Following the cotreatment of hCPCs with MHY-1684 (0.1, 1, and 10 *μ*M) and 25 mM D-glucose for 72 h, cell viability was assessed. Values are the mean ± S.E.M. ^∗^ *p* < 0.05 as compared with the control group; ^#^ *p* < 0.05 as compared with the 25 mM D-(+) glucose-treated group. (c) After treatment with MHY-1684 for 24 h in 25 mM D-(+) glucose, the prosurvival-related proteins (ERK-1, AKT-1) were analyzed by Western blotting. (d) hCPCs were incubated with 25 mM D-(+) glucose for 0–72 h with or without 1 *μ*M MHY-1684 for 72 h. hCPCs were stained with annexin V/PI, and then, hCPCs were measured by flow cytometry. hCPCs were classified as viable cells (annexin V-/PI-), apoptotic cells (annexin V+/PI-, annexin V+, PI+), and dead cells (annexin V+/PI+). (e) Dead cell population is expressed as a percent of the total cell population. (f) Apoptotic cell population is expressed as a percent of the total cell population. Values are the mean ± S.E.M. ^∗^ *p* < 0.05 and ^∗∗^ *p* < 0.01 as compared with the control group; ^#^ *p* < 0.05 as compared with the group treated with 25 mM D-(+) glucose for 72 h.

To investigate whether hyperglycemia induced cell death in hCPCs, we treated hCPCs with 25 mM D-(+) glucose in a time-dependent manner. As a result, we found that this concentration of glucose induced a significant amount of hCPC cell death in 72 h (Figure 2(a)). Importantly, hCPCs cotreated with D-(+) glucose and MHY-1684 significantly attenuated hyperglycemia-induced cell death (Figure 2(b)). Furthermore, we also observed an increase in the expression of proliferation-related markers including p-ERK and p-AKT (Figure 2(c)), suggesting that MHY-1684 might promote cell proliferation.

### 3.4. Cytoprotective Effect of MHY-1684 on Hyperglycemia-Induced Apoptosis in hCPCs

To investigate whether hyperglycemia-induced cell death was caused by hyperglycemia-induced apoptosis, we evaluated hCPC cell death via annexin V/PI staining. As shown in Figure 2(d), the high glucose condition significantly increased the percentage of dead cells in the hCPC population. In contrast, pretreatment of hCPCs with MHY-1684 and high glucose for 72 h significantly attenuated the hyperglycemia-induced hCPC cell death (Figures 2(d)–2(f)).

### 3.5. MHY-1684 Attenuates Mitochondrial ROS Generation

**Figure 3:**
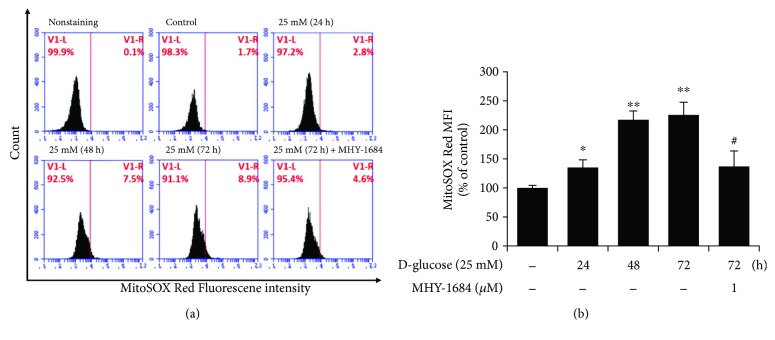
MHY-1684 attenuates mitochondrial ROS generation. (a) hCPCs were treated with 25 mM D-(+) glucose for 0–72 h and 1 *μ*M MHY-1684 for 72 h. Mitochondrial ROS were labeled with MitoSOX Red, and hCPCs were analyzed by flow cytometry. (b) Histogram indicates median value ± S.E.M. of MitoSOX mean fluorescence intensity (MFI) when exposed to D-(+) glucose. MitoSOX-positive cells increased in a time-dependent manner when exposed to D-(+) glucose; however, when cotreated with MHY-1684, the MitoSOX-positive cells decreased significantly. Values are the mean ± S.E.M ^∗^ *p* < 0.05, ^∗∗^ *p* < 0.01 as compared with the control group, ^#^ *p* < 0.05 as compared with the group treated with 25 mM D-(+) glucose for 72 h.

Based on a previous report that hyperglycemia-induced apoptosis is caused by mitochondrial ROS [17], we investigated the effect of MHY-1684 on hyperglycemia-induced mitochondrial ROS generation. As shown in Figure 3(a), when exposed to hyperglycemia, hCPCs produced more mitochondrial ROS. Importantly, cotreatment of hCPCs with MHY-1684 and D-(+) glucose significantly decreased mitochondrial ROS (Figures 3(a) and 3(b)), indicating that MHY-1684 might protect hCPCs from apoptotic cell death by blocking mitochondrial ROS generation.

### 3.6. MHY-1684 Attenuates Mitochondrial Fission via Regulating Fission/Fusion-Related Proteins

**Figure 4:**
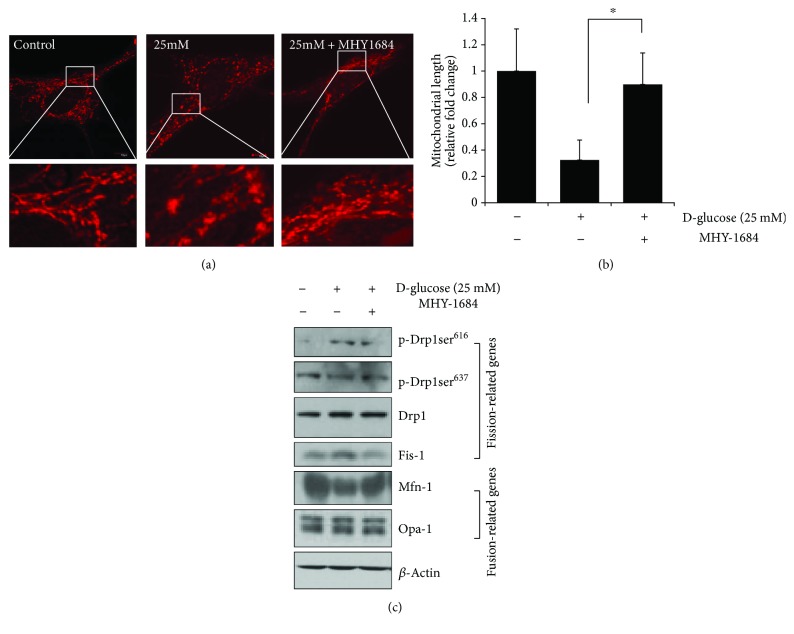
MHY-1684 attenuates mitochondrial fission via regulating fission/fusion-related proteins. (a) Confocal image of hCPCs stained with MitoTracker Red CMXRos after cotreatment with 1 *μ*M MHY-1684 and 25 mM D-(+) glucose for 72 h. (b) Mitochondrial length was analyzed when hCPCs were incubated with 1 *μ*M MHY-1684 after being exposed to 25 mM D-(+) glucose. Results are presented as means ± SD. ^∗^ *p* < 0.05 versus 25 mM D-(+) glucose. (c) Expression of the mitochondrial fragmentation-related marker Fis1, Drp1, OPA1, and Mfn1 when incubated with 1 *μ*M MHY-1684 after being exposed to 25 mM D-(+) glucose.

To examine the effect of MHY-1684 on hyperglycemia-induced mitochondrial fragmentation [20], we observed mitochondria morphological changes following hCPC treatment with glucose and MHY-1684. As shown in Figure 4(a), total mitochondrial length decreased significantly when cells were exposed to 25 mM D-(+) glucose. In contrast, cotreatment of hCPCs with 1 *μ*M MHY-1684 and 25 mM D-(+) glucose for 72 h significantly attenuated mitochondrial fragmentation (Figures 4(a) and 4(b)). To investigate the molecular mechanism by which this attenuation occurs, we next examined the expression of mitochondrial dynamics-related proteins including both the fission-associated Drp1 and Fis-1 and the fusion-associated Mfn-1 and Opa-1 by Western blotting. Notably, the activation of both Drp1 (phosphorylation at the site of Ser616) and Fis-1 is drastically attenuated when exposed to high concentrations of D-(+) glucose. Interestingly, the phosphorylation of Drp1 at Ser637, which is an inhibitory signal for mitochondrial fusion, is restored following hCPC treatment with MHY-1684. In contrast, when hCPCs are treated with MHY-1684, the expression of Mfn-1 is significantly restored, suggesting that treatment with MHY-1684 might affect the activation and inhibition of mitochondrial dynamics-related signaling and mitochondrial function in response to ROS stress (Figure 4(c)).

### 3.7. Cytoprotective Effect of MHY-1684 on hCPC Tube-Forming Capacity during Hyperglycemia

**Figure 5:**
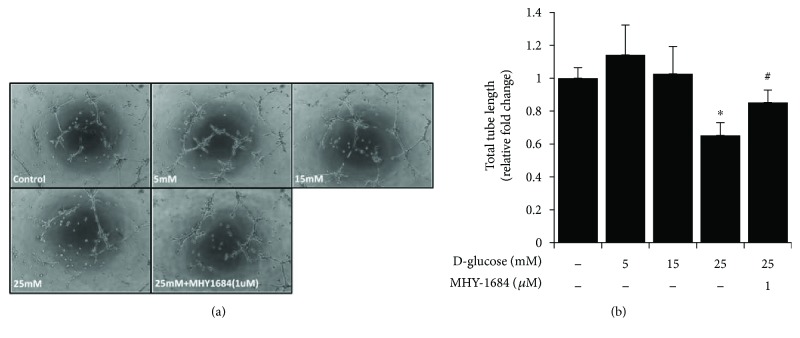
MHY-1684 rescues the tube-forming capacity of hCPCs during hyperglycemia. (a) The tube formation ability of hCPCs treated with 1 *μ*M MHY-1684 and 25 mM D-(+) glucose for 72 h. The ability for hCPCs to form capillary-like structures in vitro was evaluated using a Matrigel tube formation assay. (b) Quantification of the total length of tube-like structures. ^∗^ *p* < 0.05 as compared with the control group, ^#^ *p* < 0.05 as compared with the group treated with 25 mM D-(+) glucose for 72 h.

To determine whether pretreatment with MHY-1684 enhances the angiogenic bioactivity of hCPCs in response to hyperglycemia, we examined the tube-forming potential of MHY-1684-treated hCPCs. The tube-forming capacity decreased significantly in the presence of 25 mM D-(+) glucose. However, the hyperglycemia-reduced tube-forming capacity of hCPCs was rescued significantly when cotreated with MHY-1684 (Figures 5(a) and 5(b)), suggesting that MHY-1684 improves the angiogenic function of hCPCs during hyperglycemia.

## 4. Discussion

Although the clinical impact of CPC-based therapies is emerging [26] and the therapeutic effect of CPCs in ischemic cardiovascular disease models seems to be clearly demonstrated, there is a limitation based on the quality and quantity of resident CPCs that can be used for therapeutic applications in a clinical setting. The medical community is limited because patient-derived CPCs possess reduced therapeutic bioactivities due to multiple risk factors including age, smoking, diabetics, and hyperglycemia. In order to achieve a significant therapeutic effect from transplanted CPCs, there has been an increased focus on the discovery of novel function-modulating factors including ROS scavengers [13–15]. In this report, we identified a novel antioxidant, MHY-1684, which enhanced hCPC bioactivity against ROS-related diabetic cardiomyopathy. Interestingly, short-term treatment with MHY-1684 in ex vivo-expanded CPCs attenuated hyperglycemia-induced mitochondrial fragmentation and cell death. This suggests that MHY-1684 might be a novel priming agent for CPC-based therapies for diabetic cardiomyopathy via reducing mitochondrial ROS production and regulating mitochondrial dynamics.

Mitochondria are energy-producing organelles that are mobile and harbor dynamic network structures. Maintaining mitochondrial dynamics is an important part of tissue homeostasis in that disordered mitochondrial dynamics is associated with various diseases [27]. Existing evidence also suggests that mitochondrial fragmentation occurs during hyperglycemia and thereby causes hCPC dysfunction [17]. Our results support the importance of the proper modulation of hyperglycemia-related mitochondrial dynamics. Pretreatment of hCPCs with MHY-1684 dramatically reduced hyperglycemia-related mitochondria fission via attenuating the activation of Drp-1 and Fis-1. This was demonstrated by Western blotting using both p-Drp-1^616^ and Fis-1 antibodies (Figures 4(a) and 4(b)). In contrast, both p-Drp-1^637^ and the fusion-related protein, Mfn-1, became reversely activated during hyperglycemia. These results suggest that the disordered mitochondrial dynamics is partially restored by MHY-1684 (Figure 4(c)).

Mitochondrial ROS are formed naturally as a metabolic by-product in cells, and they play crucial roles in cell signaling, proliferation, homeostasis, and cell death [28]. In the clinical setting, hyperglycemia causes the overproduction of ROS, which disrupts cell structures and causes cell death [29], leading to cardiomyopathy. To reduce the effects of hyperglycemia such as the development of diabetic cardiomyopathy, mitochondrial ROS needs to be controlled. Therefore, our novel ROS scavenger, which enhances cellular function including tube formation and hCPC cell survival, might be therapeutic in a CPC-based therapy against diabetic cardiomyopathy. Specific modulation of ROS-related hyperglycemia is also an important issue because hyperglycemia inhibits the angiogenic capacity of transplanted cells and resident stem/progenitor cells and suppresses cell homing by regulating the ERK1/2 pathway [9, 21]. In our current study, we demonstrated that the attenuation of ROS by MHY-1684 activates the ERK1/2 and AKT signaling pathways. Multiple studies support the fact that ROS overproduction suppresses ERK1/2 and AKT signaling and eventually leads to cell death [30, 31], suggesting that augmented ERK1/2 and AKT signaling partially affects the rescue process mediated by MHY-1684 in hyperglycemia-induced diabetic cardiomyopathy.

## 5. Conclusion

**Figure 6:**
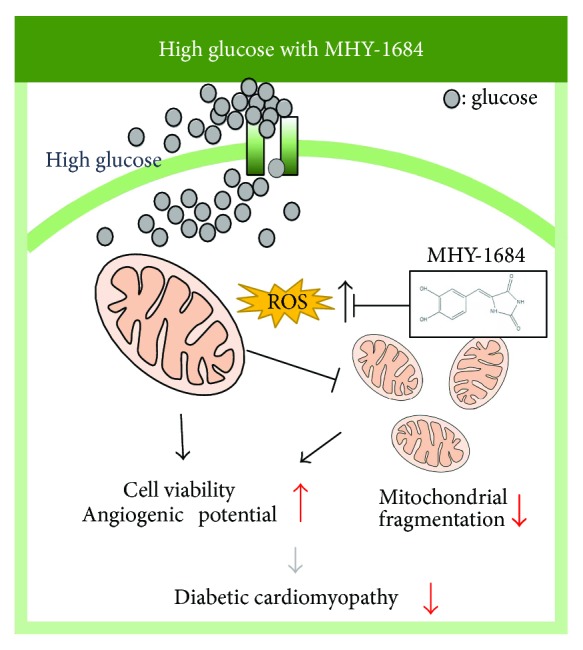
Proposed working model. MHY-1684 plays a critical role in ROS generation, restores the angiogenic potential, and reduces cell death of hCPCs via regulating mitochondrial dynamics.

Our results demonstrated that MHY-1684 enhances hCPC cellular function against ROS and hyperglycemia. Importantly, MHY-1684 attenuated mitochondrial ROS generation during hyperglycemia and significantly reduced mitochondrial fragmentation via regulating Drp-1 and Fis-1. MHY-1684 also decreased ROS-induced cell death (Figure 6). In conclusion, the novel compound, MHY-1684, is an ROS scavenger that might be an effective therapeutic agent for the CPC-based therapy against diabetic cardiomyopathy.

## Data Availability

The data used to support the findings of this study are available from the corresponding author upon request.

## Supplementary Materials

Supplementary Materials Supplementary Table 1: ONOO^−^ (10 *μ*M) scavenging activities of chemicals (40 *μ*M). The potency of scavenging activity following the addition of authentic ONOO^−^ or SIN-1 was in the following order: MHY-1684 > penicillamine > resveratrol. MHY-1684 showed the strongest effect for scavenging ONOO^−^ of chemicals.
