# Supplementary material for: Cytoprotective Roles of a Novel Compound, MHY-1684, against Hyperglycemia-Induced Oxidative Stress and Mitochondrial Dysfunction in Human Cardiac Progenitor Cells
Source: Oxid Med Cell Longev. 2018 May 30;2018:4528184. doi: 10.1155/2018/4528184 (PMC5998189; doi:10.1155/2018/4528184)
Supplement: Supplementary Materials — Supplementary Table 1: ONOO− (10 μM) scavenging activities of chemicals (40 μM). The potency of scavenging activity following the addition of authentic ONOO− or SIN-1 was in the following order: MHY-1684 > penicillamine > resveratrol. MHY-1684 showed the strongest effect for scavenging ONOO− of chemicals. [file 4528184.f1.pdf]

Supporting Information

Table S1. $ONOO^-$ (10μM) scavenging activities of chemical (40μM)

Common name	Molecular Formula	Authentic $ONOO^-$(%)	$ONOO^-$ from Decomposition of SIN-1(%)
MHY-1684	$C_{10}H_9N_2O_4$	98.5±0.56	94.75±2.28
Pencilamine	$C_5H_{11}NO_2S$	93.83±0.83	92.88±0.21
Resveratrol	$C_{14}H_{12}O_3$	18.03±1.20	36.80±16.44

Data are mean ± for triplicate measurements.
